# Random Structure Searching with Orbital-Free Density Functional Theory

**DOI:** 10.1021/acs.jpca.0c11030

**Published:** 2021-02-15

**Authors:** William C. Witt, Benjamin W. B. Shires, Chuin Wei Tan, Wojciech J. Jankowski, Chris J. Pickard

**Affiliations:** † Department of Materials Science and Metallurgy, University of Cambridge, Cambridge, U.K.; ‡ Downing College, University of Cambridge, Cambridge, U.K.; ¶ Trinity College, University of Cambridge, Cambridge, U.K.; § Advanced Institute for Materials Research, Tohoku University, Sendai, Japan

## Abstract

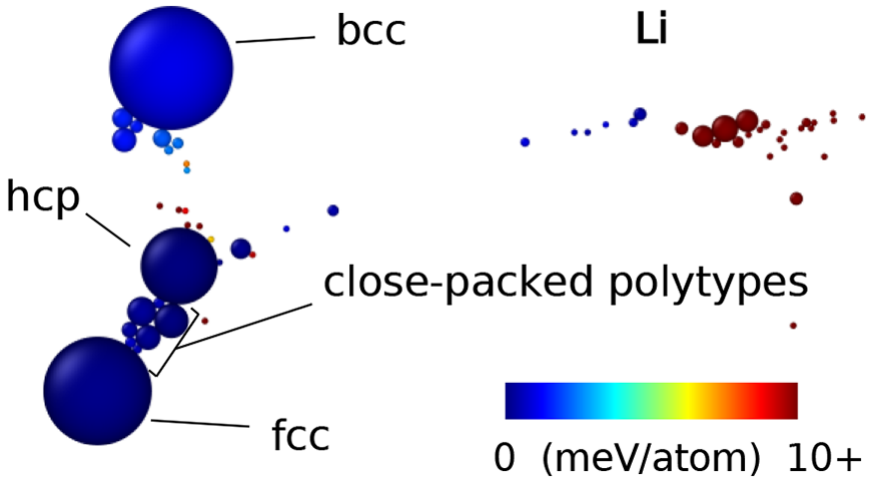

The properties of a material depend on how its atoms are arranged, and predicting these arrangements from first principles is a longstanding challenge. Orbital-free density functional theory provides a quantum-mechanical model based solely on the electron density, not individual wave functions. The resulting speedups make it attractive for random structure searching, whereby random configurations of atoms are relaxed to local minima in the energy landscape. We use this strategy to map the low-energy crystal structures of Li, Na, Mg, and Al at zero pressure. For Li and Na, our searching finds numerous close-packed polytypes of almost-equal energy, consistent with previous efforts to understand their low-temperature forms. For Mg and Al, the searching identifies the expected ground state structures unambiguously, in addition to revealing other low-energy structures. This new role for orbital-free density functional theory—particularly as continued advances make it accurate for more of the periodic table—will expedite crystal structure prediction over wide ranges of compositions and pressures.

## Introduction

Materials physicists have long sought efficient methods for predicting the structures of atoms in materials. The field can now claim many successes,^1−3^ propelled by steady increases in computing power. One fruitful strategy combines density functional theory (DFT)^4−7^ and random structure searching.^1,8^ The former provides reliable estimates of the energy (or enthalpy) of an arrangement of atoms, while the latter explores possible configurations. The procedure begins with randomly generated structures, perhaps having preselected symmetries or constrained by simple heuristics, which are then relaxed to local minima (or stationary points) in the energy landscape. This approach, while pragmatic and effective, is limited by the computational expense of conventional Kohn–Sham density functional theory (KSDFT).^5^ For example, metals requiring dense Brillouin zone sampling pose a challenge because poor sampling leads to overly rugged landscapes. Faster DFT calculations would facilitate study of numerous materials of practical interest, such as complex phases of intermetallic alloys.

Orbital-free density functional theory (OFDFT)^9−12^ achieves large speedups over orbital-based KSDFT. Accordingly, for a fixed computational resource and time period, OFDFT can drive more geometry optimizations than KSDFT, even if the systems are relatively small, accelerating the overall structure searching task. In principle, the methods are equally rigorous, but in practice OFDFT is typically less accurate. At present, OFDFT achieves near-KSDFT accuracy only for free-electron-like metals and some semiconductors, but new advances continue to widen its applicability. Importantly, to succeed in random structure searching, OFDFT need only *locate* the most relevant structures. The results of such a search are easily validated and refined.

## Methods

To find a system’s ground state with OFDFT, one expresses all energy contributions as functionals of the electron density and then varies the density to minimize the total energy.^13,14^ Conventional KSDFT is similar, except the noninteracting kinetic energy, *T*_*s*_, is calculated from the Kohn–Sham orbitals, *not* with a density functional. Therefore, for OFDFT to achieve results that agree with the orbital-based approach, the first requirement is an accurate density functional approximation of the form *T*_*s*_[*n*].

A second potential source of error for OFDFT enters when pseudopotentials are used to represent core electrons and nuclei. OFDFT requires strictly local pseudopotentials since nonlocal pseudopotentials rely on orbital information. This restriction is not severe for free-electron-like metals, as we demonstrate below.

To conduct OFDFT-driven random structure searching, we relaxed 1000 random structures for each of Li, Na, Mg, and Al. We produced the initial structures with the AIRSS software,^1,8^ constraining the initial unit cells to have volumes within 5% of the expected equilibrium volumes (see below for more details). The structural relaxations were challenging tests for OFDFT, requiring it to perform reliably for many diverse configurations drawn from across the corresponding energy landscapes.

In the rest of this section, we provide theoretical and computational details, including modifications to established kinetic energy functionals that improve their robustness for random search. Readers uninterested in these details may skip directly to the Results and Discussion, where we analyze the outcomes of the structure searching.

### Approximating the Kinetic Energy with a Density Functional

We begin by expressing the noninteracting kinetic energy in the form of a sum
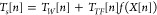
1where the first term is the Weizsäcker kinetic energy,^15^
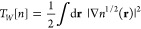
2which is exact for single-orbital systems. The second term is the excess Pauli kinetic energy,^16,17^ expressed as the product of the Thomas-Fermi kinetic energy,^18,19^*T*_*TF*_, and an enhancement factor, *f*(*X*). The Thomas–Fermi functional, which is exact for a free electron gas, is
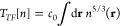
3with *c*_0_ = (3/10)(3π^2^)^2/3^. The Pauli kinetic energy is always nonnegative, and it is typically nonzero as a consequence of the Pauli Exclusion Principle.

A few preliminary comments on the enhancement factor function *f*(*x*) are appropriate. First, if *f*(*x*) is invertible, then eq 1 can be considered exact, with the functional *X* defined as X = *f*^–1^([*T*_*s*_ – *T*_*W*_]/*T*_*TF*_). For a given invertible *f*(*x*), one is left to approximate *X*.

Second, the choice *f*(*x*) = 1 + *x* allows recovery of an important class of interrelated kinetic energy functionals used widely for materials research, including those of Wang and Teter;^20^ Perrot;^21^ Smargiassi and Madden;^22^ and Wang, Govind, and Carter.^23^ However, these Wang–Teter-style approximations can violate the *T*_*s*_ ≥ *T*_*W*_ constraint, at times to catastrophic effect. In fact, Blanc and Cancès showed that such approximations are unbounded from below,^24^ providing a mathematical origin for instabilities observed in practice. Therefore, because *T*_*W*_ is nonnegative, strict imposition of *T*_*s*_ ≥ *T*_*W*_ would have a stabilizing effect.

Finally, by judicious choice of *f*(*x*), one may devise new approximations that reduce to the original Wang–Teter-style functionals when appropriate, but also obey the *T*_*s*_ ≥ *T*_*W*_ constraint. The nonnegative function *f*(*x*) = *e*^*x*^ is especially suitable because *e*^*x*^ ≈ 1 + *x* when *x* is small. We adopt this choice throughout, but it is far from the only possibility. The exponential-stabilized functionals facilitate the geometry optimizations required for random structure searching, while retaining the best features of the original approximations.

### Approximating *X*[*n*]

Drawing on the earlier work,^20−23^ we approximate *X*[*n*] as
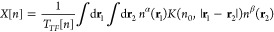
4

The form of the weight function, *K*(*n*_0_, |**r**|) is deduced below; for now, we specify only that it depends on a uniform electron density *n*_0_ and a distance between points in space. For crystals, amorphous solids, and liquids, there is a natural choice for *n*_0_, the average electron density, which we adopt throughout. For other systems with regions of vacuum, more care is required when choosing *n*_0_.

For the limiting case of a free electron gas, the second derivative of *T*_*s*_ is known in analytical form from perturbation theory:^25^
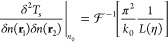
5where  denotes inverse Fourier transformation, *k*_0_ = (3π^2^*n*_0_)^1/3^, η = *k*/(2*k*_0_), *k* is the length of the reciprocal space vector **k**, and
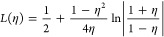
6

It proves useful to incorporate the condition of eq 5 into approximate density functionals, particularly for nearly free-electron metals. This information is sufficient for determining the weight function in eq 4:
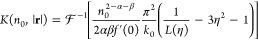
7

Importantly, if *f*′(0) = 1, then the weight function in eq 7 reduces to the “density-independent” weight function from ref (23).

We are now able to fully specify the Wang–Teter, Perrot, Smargiassi–Madden, and Wang–Govind–Carter functionals in our notation: they are defined by eqs 1, 4, and 7, with *f*(*x*) = 1 + *x* and different choices for α and β. For the Wang–Teter functional, α = β = 5/6, as inspired by the Thomas–Fermi functional; for the Perrot functional, α = β = 1, exactly as in perturbation theory; for the Smargiassi–Madden functional, α = β = 1/2, producing the correct semiclassical behavior for the slowly varying limit; and, for the Wang–Govind–Carter functional, , yielding correct asymptotic behavior for both the slowly varying and rapidly varying limits. These properties do not necessarily carry over to the exponential-stabilized functionals.

### Kinetic Potential and Kinetic Stress

The kinetic potential for the approximation defined by eqs 1, 4, and 7 is
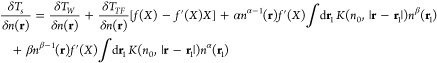
8with *δT*_*W*_/*δn*(**r**) = −(1/2)(∇^2^*n*^1/2^)/*n*^1/2^ and *δT*_*TF*_/*δn*(**r**) = (5/3)*c*_0_*n*^2/3^. The associated contribution to the stress tensor is
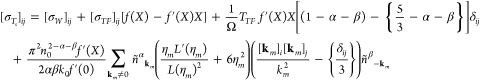
9where Ω is the cell volume, δ_*ij*_ is the Kronecker delta, , [σ_*TF*_]_*ij*_=–2/(3Ω)*T*_*TF*_δ_*ij*_, the {**k**_*m*_} are the reciprocal lattice vectors, and
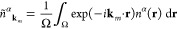
10

The terms in curly braces in eq 9 are necessary if *n*_0_ is set to the average electron density (and therefore adjusts as the cell is stretched infinitesimally), but should be omitted if *n*_0_ is treated as a fixed external parameter. If *f*(*x*) = 1 + *x*, eq 9 is equivalent to the associated formulas given in ref (14).

### Other Approximations

Our choice of functional for the noninteracting kinetic energy was motivated by pragmatism: we began with a form known to perform well for nearly free-electron metals and generalized it to enforce nonnegativity in the Pauli kinetic energy. This approximation encodes sufficient physics for accurate treatment of the low-energy structures (and suitably robust treatment of higher-energy structures) of Li, Na, Mg, and Al, all while retaining computational efficiency.

However, kinetic energy functional development is an active area of research,^26−32^ and numerous approximate functionals, having various desirable features, are available.^9−12^ Some, like ours, enforce the *T*_*s*_ ≥ *T*_*W*_ constraint by construction.^28,33−35^ Others would perform better than ours for semiconductors.^36−38^ A few recent papers have even utilized randomly generated crystal structures or clusters, but without relaxation, to assess the performance of new kinetic energy functionals.^29,31,32^ For future OFDFT-driven random structure searching, it will be advantageous to adopt some of these many approximations.

## Computational Details

We used the AIRSS package^1,8^ to generate the initial structures, producing 1000 random structures for each of Li, Na, Mg, and Al. Each structure had between 3 and 12 atoms, and we generated 100 of each size. We constrained the initial unit cells to have volumes within 50% of 19.7, 35.9, 22.0, and 15.6 Å^3^/atom for Li, Na, Mg, and Al, respectively, and to have minimum distances between atoms of 1.5 Å.

We conducted both OFDFT and KSDFT calculations, with the latter serving to validate and refine predictions of the former. In all results reported in the main paper, we employed the Perdew–Burke–Ernzerhof (PBE) generalized gradient approximation^39^ to account for electronic exchange and correlation. In the Supporting Information, we provide some analogous results obtained with the local density approximation (LDA)^40^ and PBEsol^41^ functionals. Computational settings were chosen to converge predicted energies to within 1 meV/atom.

We completed the OFDFT calculations with a new version of the PROFESS code,^14,42,43^ using the local pseudopotentials for Li, Na, Mg, and Al reported in refs (44, 45, 46, and 46), respectively. For structure searching, we used a plane wave cutoff of 800 eV for the electron density; for all other OFDFT calculations, we used 2000 eV. Because of the demands of random search, we coupled PROFESS to the Atomic Simulation Environment^47^ to make use of the latter’s superior geometry optimization tools. To minimize forces and stresses, we used the BFGS with line search option with the ExpCellFilter feature for simultaneous variation of the cell and ion degrees of freedom (see ref (48) for details of the method). We restarted the minimization every 3 steps for 3 iterations, then every 20 steps until convergence—the restarts allow the grid shape to adjust to changes in the cell shape. Even with the exponential-stabilized kinetic energy functionals, some structure optimizations still failed, typically because the cell became extremely skewed or because the crystal began to dissociate. These failures are not especially consequential—they occur in KSDFT-driven random structure searching too, although less frequently—and the candidate structures are simply rejected.

We performed the KSDFT calculations with CASTEP,^49^ using either the local pseudopotentials mentioned previously or the C19 set of ultrasoft nonlocal pseudopotentials. The latter are presumed more accurate. Each calculation used a plane wave cutoff for the orbitals of 1000 eV and Brillouin zone sampling with Monkhorst–Pack^50^ grids having maximum distance between *k*-points of 2π × 0.015 Å^–1^. The CASTEP geometry optimizations used the default LBFGS method.

### Testing the Modified Kinetic Energy Functionals

For benchmarking, we used OFDFT and KSDFT to predict the equilibrium volumes, relative energies, and bulk moduli for the body-centered cubic (bcc), cubic diamond (cd), face-centered cubic (fcc), hexagonal close-packed (hcp), and simple cubic (sc) structures. The relative energies appear in Table 1 and the remaining data are in Tables S1 and S2 in the Supporting Information. Each table contains eight columns of OFDFT predictions generated with the four Wang–Teter-style functionals and their exponential-stabilized forms. They also contain KSDFT predictions in columns labeled KS-L and KS-NL for local and nonlocal pseudopotentials, respectively. Good agreement between the OFDFT columns and the KS-L column indicates that the kinetic energy functional is suitable, whereas good agreement between the KS-L and the KS-NL columns indicates that the local pseudopotential is suitable.

**Table 1:** Relative Energies (meV/atom) for Several Elements and Crystal Structures as Predicted by OFDFT Using Local Pseudopotentials and Eight Variations of the Same Kinetic Energy Functional (Wang–Teter, Perrot, Smargiassi–Madden, and Wang–Govind–Carter, along with Their Exponential-Stabilized Forms) and as Predicted by KSDFT Using both Local Pseudopotentials (KS-L) and Nonlocal Pseudopotentials (KS-NL)^a^

**Li**										
	WT	WT-e	P	P-e	SM	SM-e	WGC	WGC-e	KS-L	KS-NL
fcc	0	0	0	0	0	0	0	0	0	0
hcp	0	0	0	0	0	0	0	0	0	0
bcc	1	1	1	1	1	1	1	1	1	2
sc	151	151	150	149	156	155	151	151	152	120
cd	429	432	426	429	437	*441*	429	432	428	516

**Na**										
	WT	WT-e	P	P-e	SM	SM-e	WGC	WGC-e	KS-L	KS-NL
fcc	0	0	0	0	0	0	0	0	0	0
hcp	0	0	0	0	0	0	0	0	0	0
bcc	1	1	1	1	1	1	1	1	1	1
sc	109	109	108	108	112	112	109	109	110	119
cd	307	313	307	312	307	314	307	312	306	337

**Mg**										
	WT	WT-e	P	P-e	SM	SM-e	WGC	WGC-e	KS-L	KS-NL
hcp	0	0	0	0	0	0	0	0	0	0
fcc	11	10	9	8	20	16	11	10	14	13
bcc	27	26	27	26	27	25	28	26	29	29
sc	*392*	*393*	*376*	*379*	*465*	*454*	*394*	*395*	410	381
cd	*840*	*907*	*799*	*875*	*884*	*972*	*829*	*902*	854	774

**Al**										
	WT	WT-e	P	P-e	SM	SM-e	WGC	WGC-e	KS-L	KS-NL
fcc	0	0	0	0	0	0	0	0	0	0
hcp	18	16	*14*	*14*	31	27	18	17	25	32
bcc	73	70	*61*	*59*	*113*	*102*	74	71	80	95
sc	*312*	*356*	*310*	*351*	*299*	*367*	*307*	*352*	335	371
cd	*791*	*1022*	–	*929*	*724*	*1058*	*746*	*997*	723	747

a OFDFT estimates differing from the corresponding KS-L energy by more than 10 meV/atom are italicized.

The most important pattern in the relative energies (Table 1) is that the phase orderings are correct for all cases. For Li and Na, the various methods yield essentially identical predictions for the denser, lower-energy structures (bcc, fcc, and hcp), while, for the more open structures (cd and sc), the OFDFT and KS-L predictions agree to within a few meV/atom and differ by slightly more from the KS-NL predictions. A somewhat similar pattern is apparent for Mg and Al, although there is more variation in the OFDFT data. In a few cases (particularly for some rather unphysical open structures of Al), the exponential-stabilized functionals yield notably less accurate energies than their counterparts, but the origin of this result is unclear. In general, the relative energies predicted by the stabilized functionals are greater than or equal to those for the corresponding original functionals (and never more than 2 meV/atom less than those for the original functionals), likely because the former enforce the lower bound on the Pauli kinetic energy whereas the latter do not.

**Figure 1:**
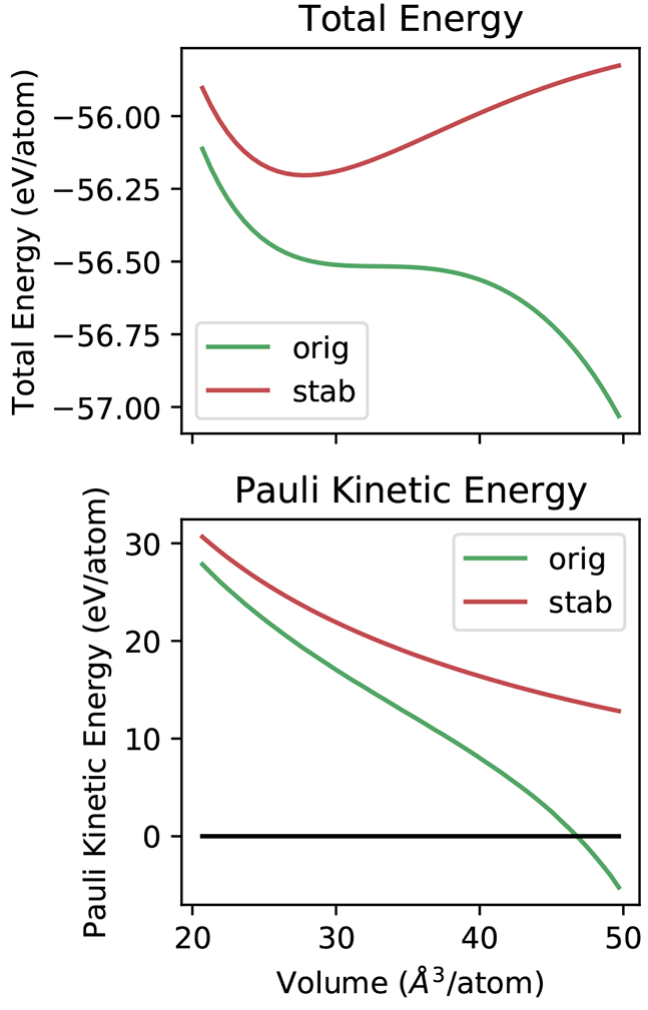
For the original and exponential-stabilized Perrot functionals: total energy (top) and Pauli kinetic energy (bottom) as a function of volume for Al in the diamond structure. The total energy curve for the original functional has no minimum, in part because of negative Pauli kinetic energies for large volumes. The exponential-stabilized functional correctly enforces nonnegativity of the Pauli kinetic energy, and it does yield a minimum in the total energy. Clearly the latter would be more stable for random structure searching.

One unusual feature of the results in Tables 1, S1, and S2 is the absence of data in the Perrot columns for the cd structure of Al. The explanation is clear from Figure 1: for the original Perrot functional, the total energy curve has no minimum as a function of volume, partly because the Pauli kinetic energy becomes unphysically negative. In contrast, the exponential-stabilized Perrot functional yields a total energy curve with the expected shape. This example illustrates a general point. Although the other Wang–Teter-style functionals exhibit more sensible behavior for this case, they are still susceptible to the instabilities outlined by Blanc and Cancès. Exponential stabilization resolves this issue by enforcing nonnegativity of the Pauli kinetic energy.

The main message of these benchmarks is that both the original and the exponential-stabilized approximations, for all four Wang–Teter-style functionals, yield nearly identical predictions for the denser, lower-energy structures we would expect to find during structure searching. Ultimately, we chose the exponential-stabilized functional with Wang–Govind–Carter exponents () to obtain our main results.

## Results and Discussion

**Figure 2:**
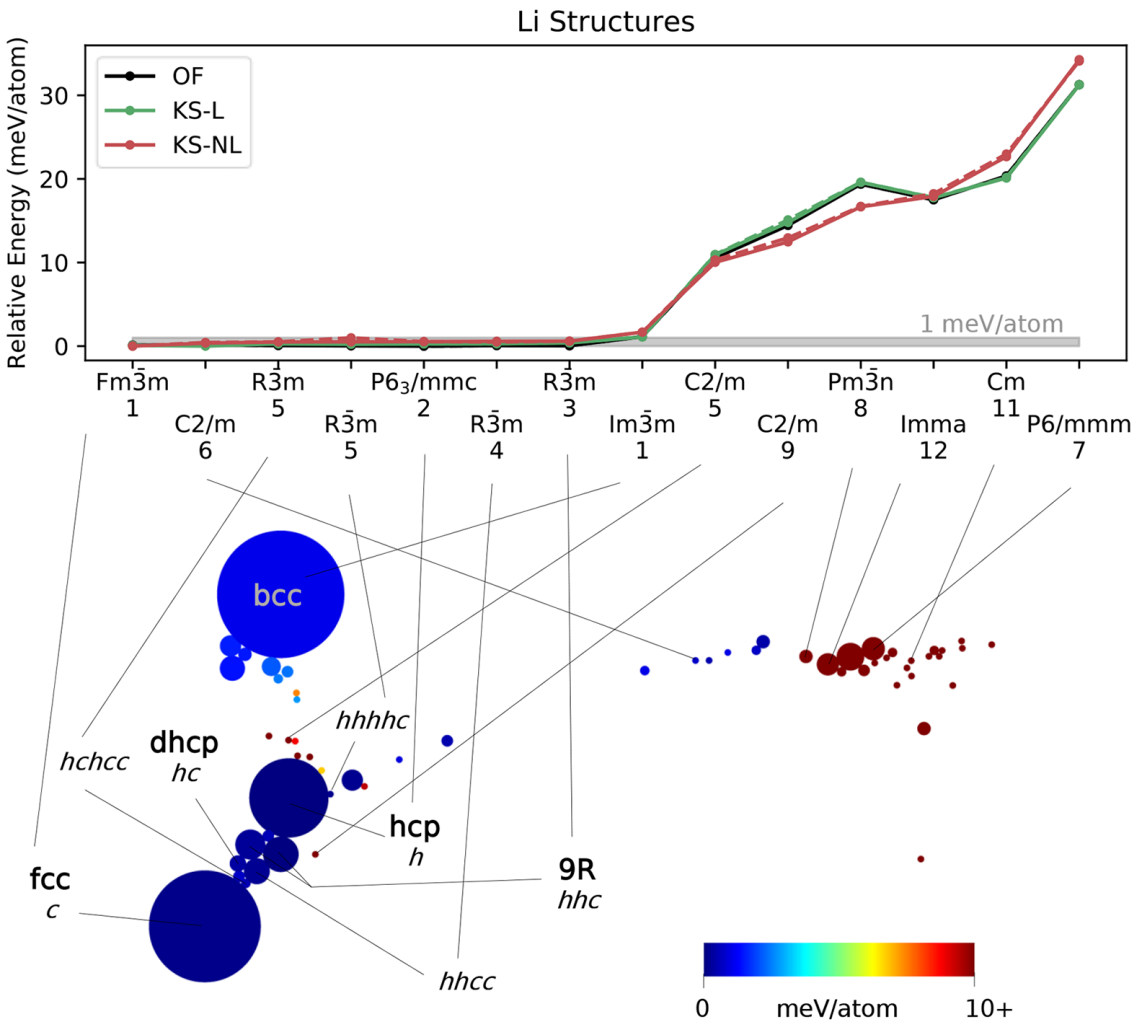
Two-dimensional visualization (bottom) of the 1000 locally stable Li structures obtained with OFDFT-driven random structure searching, showing the results of a SHEAP dimensionality reduction based on SOAP descriptors for the structures. Circle size indicates frequency of occurrence, and circle color conveys structure energy. Select structures are labeled with common names (in bold), with *hc* notation for close-packed polytypes, or with a space group paired with the number of atoms in the primitive cell. Relative energies (top) of a subset of structures computed with OFDFT (OF) and KSDFT with local or nonlocal pseudopotentials (KS-L and KS-NL, respectively). Dashed lines, where visible, show the energy for the initial structure, and solid lines show the energy after a second relaxation of the associated conventional cell.

**Figure 3:**
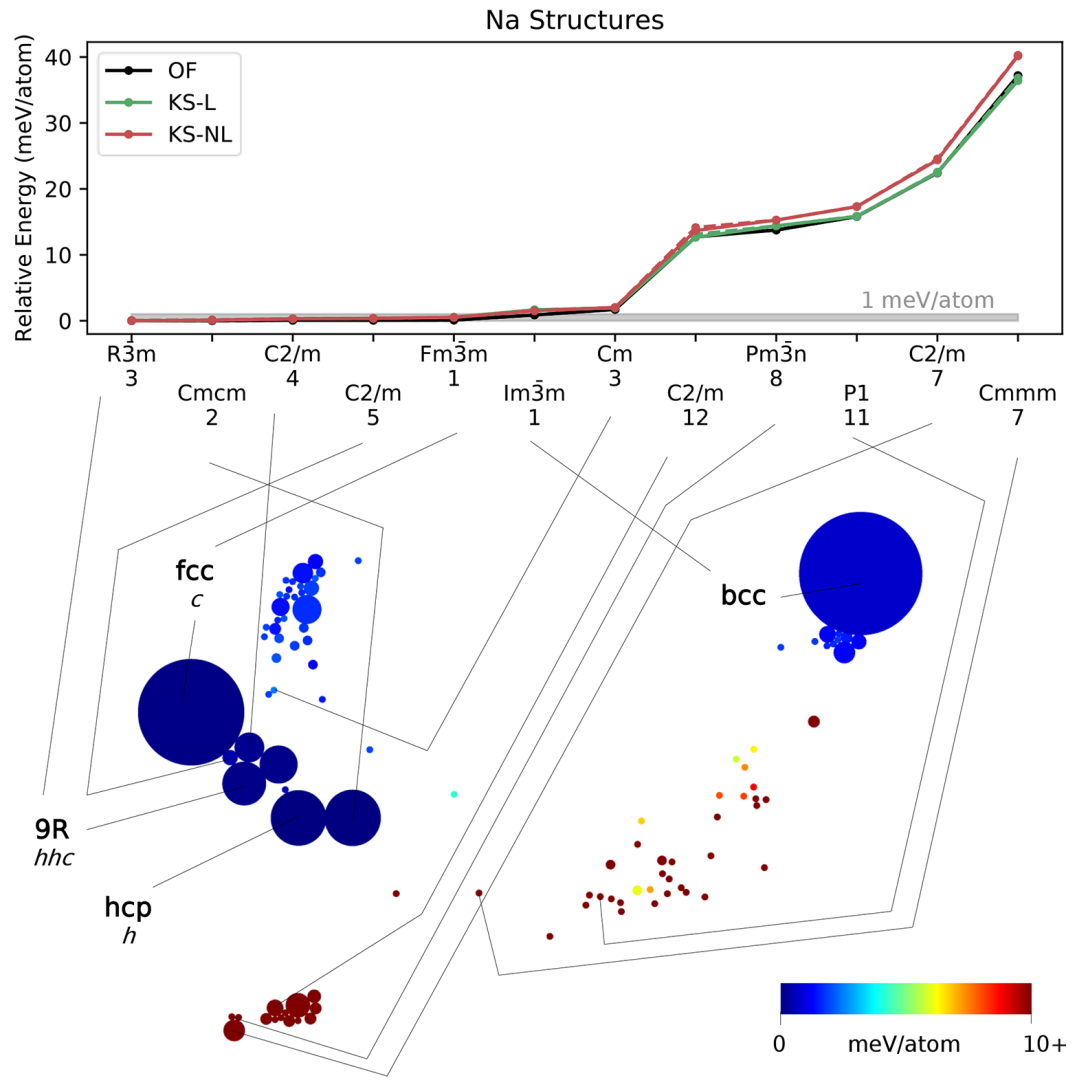
Two-dimensional visualization (bottom) of the 1000 locally stable Na structures obtained with OFDFT-driven random structure searching, showing the results of a SHEAP dimensionality reduction based on SOAP descriptors for the structures. Circle size indicates frequency of occurrence, and circle color conveys structure energy. Select structures are labeled with common names (in bold), with *hc* notation for close-packed polytypes, or with a space group paired with the number of atoms in the primitive cell. Relative energies (top) of a subset of structures computed with OFDFT (OF) and KSDFT with local or nonlocal pseudopotentials (KS-L and KS-NL, respectively). Dashed lines, where visible, show the energy for the initial structure, and solid lines show the energy after a second relaxation of the associated conventional cell.

**Figure 4:**
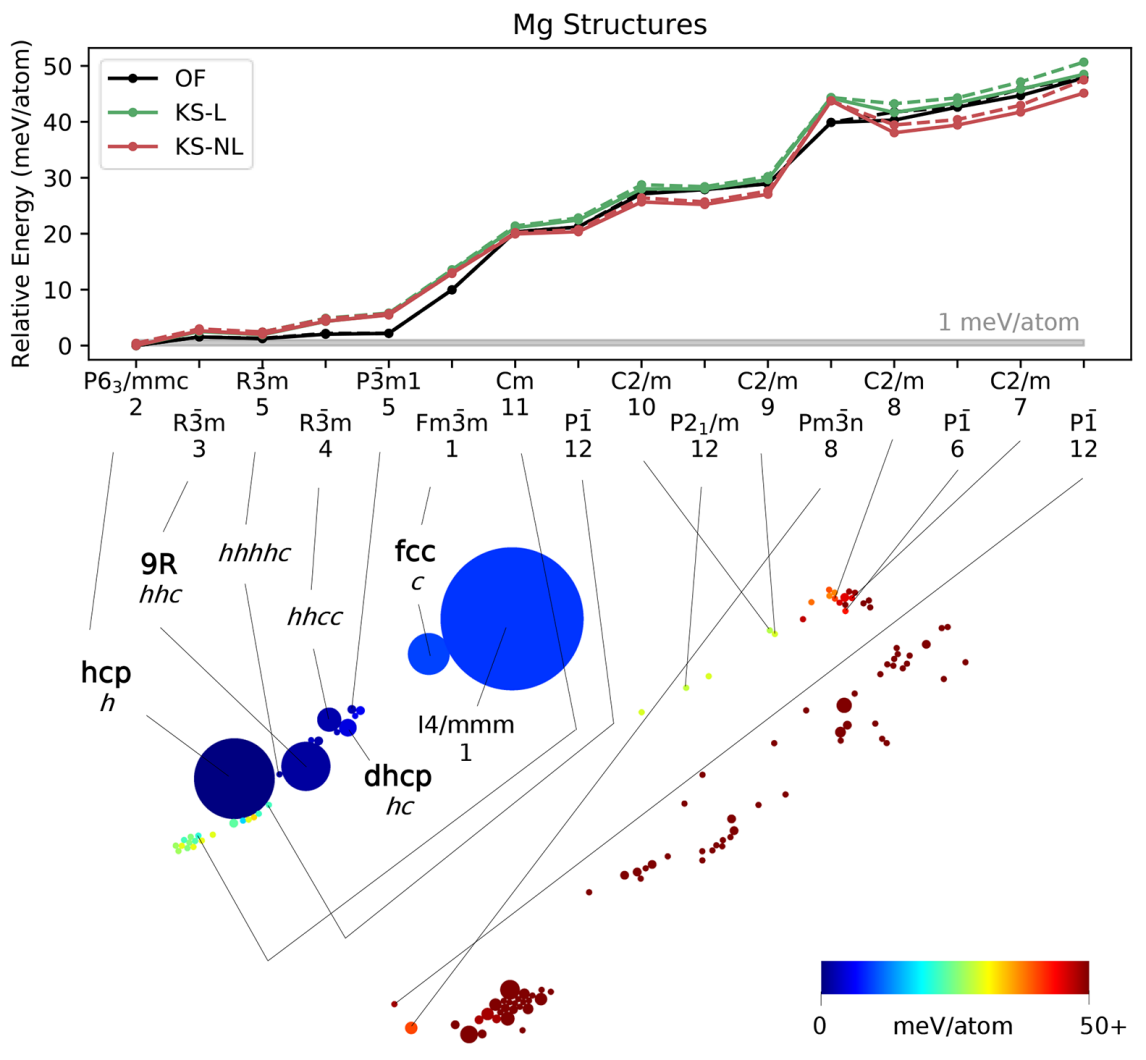
Two-dimensional visualization (bottom) of the 1000 locally stable Mg structures obtained with OFDFT-driven random structure searching, showing the results of a SHEAP dimensionality reduction based on SOAP descriptors for the structures. Circle size indicates frequency of occurrence, and circle color conveys structure energy. Select structures are labeled with common names (in bold), with *hc* notation for close-packed polytypes, or with a space group paired with the number of atoms in the primitive cell. Relative energies (top) of a subset of structures computed with OFDFT (OF) and KSDFT with local or nonlocal pseudopotentials (KS-L and KS-NL, respectively). Dashed lines, where visible, show the energy for the initial structure, and solid lines show the energy after a second relaxation of the associated conventional cell.

**Figure 5:**
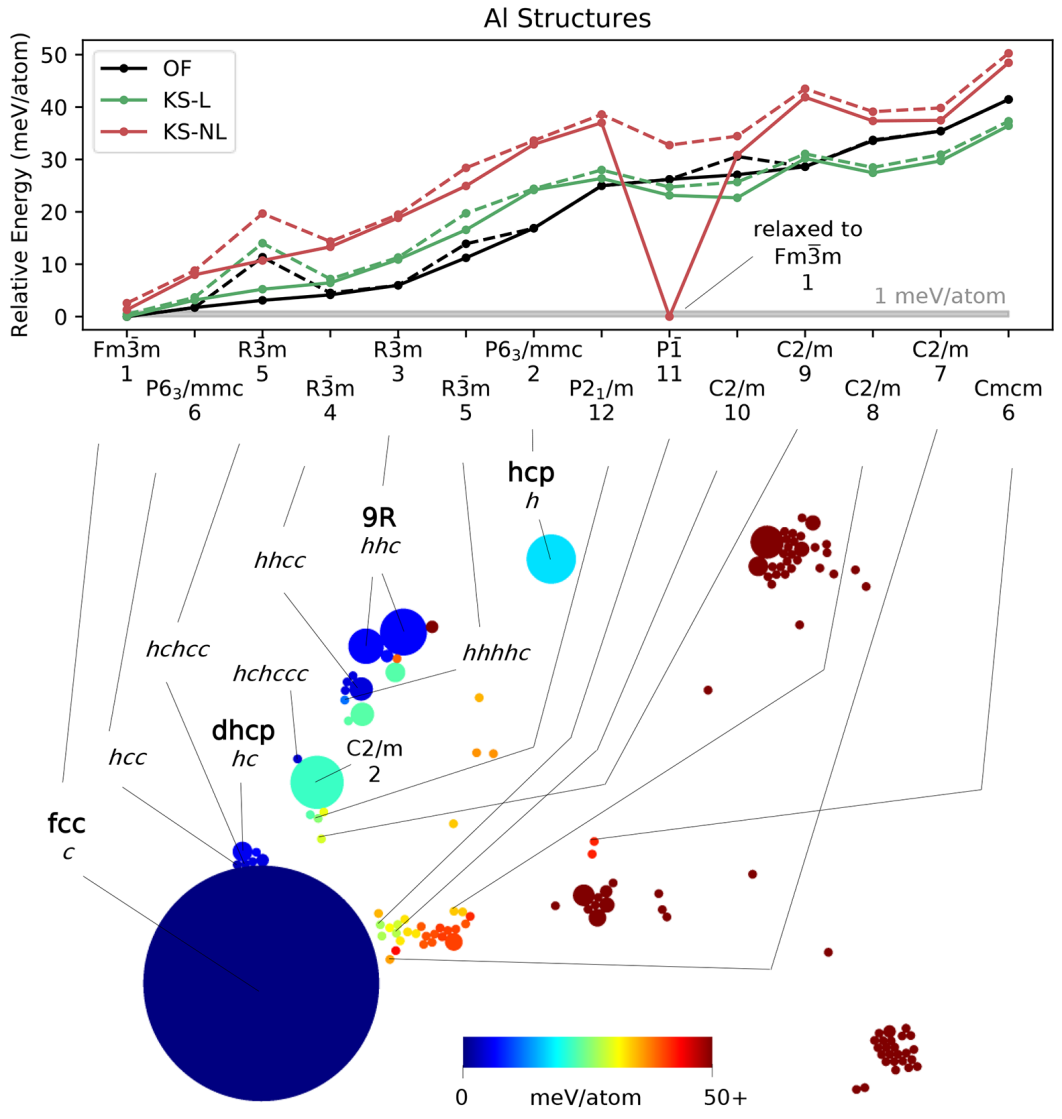
Two-dimensional visualization (bottom) of the 1000 locally stable Al structures obtained with OFDFT-driven random structure searching, showing the results of a SHEAP dimensionality reduction based on SOAP descriptors for the structures. Circle size indicates frequency of occurrence, and circle color conveys structure energy. Select structures are labeled with common names (in bold), with *hc* notation for close-packed polytypes, or with a space group paired with the number of atoms in the primitive cell. Relative energies (top) of a subset of structures computed with OFDFT (OF) and KSDFT with local or nonlocal pseudopotentials (KS-L and KS-NL, respectively). Dashed lines, where visible, show the energy for the initial structure, and solid lines show the energy after a second relaxation of the associated conventional cell.

The structure searching yielded geometries and energies of 1000 locally stable structures for each of Li, Na, Mg, and Al. To analyze these data, we began by finding primitive cells for all structures. We then generated the corresponding Smooth Overlap of Atomic Positions (SOAP)^51^ descriptors, characterizing each structure as a vector in a high-dimensional space (see the Supporting Information for details).^52^ Finally, we applied the dimensionality reduction method Stochastic Hyperspace Embedding And Projection (SHEAP) to produce two-dimensional visualizations of the structural data.^75^ In these SHEAP maps (see Figures 2–5), individual structures are represented by circles colored according to structure energy, with areas proportional to number of occurrences in the search. The axes do not have a predetermined physical interpretation, but the relative positioning and clustering across the two-dimensional space reveals structural relationships. (We also produced three-dimensional SHEAP visualizations for each element, but found no added benefit for these data sets—see the Supporting Information.)

Before remarking on the four elements separately, we note one common characteristic: their SHEAP maps all have a region of close-packed structures with fcc at one end and hcp at the other. Between these end points lie other close-packed polytypes (or distortions thereof) having close-packed planes stacked in increasingly complex sequences.^53,54^ In conventional ABC notation, fcc stacking is denoted ABC ABC···, while hcp stacking is AB AB···; however, this notation becomes burdensome in other cases. A compact *hc* notation is more suitable,^53,54^ in which hcp is represented by *h* because each layer has hcp-like stacking (identical layers above and below), and fcc is represented by *c* because each layer has fcc-like stacking (distinct layers above and below). The double hexagonal close-packed (dhcp) structure is then *hc* (replacing ABCB···), and the 9R structure becomes *hhc* (replacing ABACACBCB···). Importantly, consistent with intuition, close-packed polytypes that are clearly hcp-like, such as *hhhhc*, are found near hcp in the SHEAP maps of Figures 2–5, whereas those that are more fcc-like, such as *hchcc*, appear near fcc.

Figures 2–5 also assess the accuracy of the OFDFT predictions. Beginning with a representative subset of low-energy structures for each element, we rerelaxed each one according to the following protocol. (To obtain the subsets of structures, we used the analysis tools provided with the AIRSS software. We generated a set of distinct candidates with the command “ca -u 0.2 -r” and then eliminated those with energies greater than 50 meV/atom from the lowest-energy structure. The details of the algorithm are not essential, only that it yields an unbiased subset.)

1. Convert the primitive cell to its associated conventional cell. (Some primitive cells had skewed cell shapes that inhibited very careful relaxation.)
2. Rerelax the conventional cell with OFDFT, without symmetry constraints and using the higher plane wave cutoff. Discard the structure if it transforms significantly. (Only one structure was discarded in this manner, a rather high-energy Li structure that proved unstable.)
3. Rerelax the original conventional cell using KSDFT and the local pseudopotential, preserving the symmetry operations during the relaxation.
4. Repeat the previous step but with the corresponding nonlocal pseudopotential.

All together, the results of this procedure (Figures 2–5) establish that OFDFT, while not perfect, yields predictions in basic agreement with KSDFT for the wide range of structures located during random structure searching. These results also help disaggregate error attributable to the local pseudopotential from error attributable to the kinetic energy functional.

Finally, in the Supporting Information, we show the results of repeating the fourth step in the previous paragraph using the LDA and PBEsol exchange-correlation functionals (the latter is especially appropriate for densely packed solids). The relative energies derived with the LDA, PBE, and PBEsol approximations are almost indistinguishable, increasing trust in the predictions.

### Lithium

The most striking results for Li (Figure 2) are the numerous close-packed polytypes having low energies all within a band of 1 meV/atom, including fcc, hcp, and 9R, as well as more complex layerings like *hhcc*, *hchcc*, and *hhhhc*. Interestingly, we also located the dhcp structure a small number of times; Hutcheon and Needs, who recently performed a smaller KSDFT-driven search for Li, remarked that dhcp was notably absent from their results.^55^ Lithium’s room temperature structure, bcc, is also prominent in the Li SHEAP map; it becomes thermodynamically stabilized over fcc, hcp, and 9R at temperatures above roughly 160, 130, and 70 K, respectively.^56^

The low-temperature polytypism in Li is well-known and has been the subject of some uncertainty.^55−60^ In our calculations, several of the candidate structures differ by only tens of μeV/atom, well below our convergence threshold of 1 meV/atom. In fact, only recently was fcc established as the true ground state.^56^ Before then, other polytypes, particularly the 9R structure,^61^ were thought to be more favorable. One explanation is kinetics:^56^ the bcc–fcc transition at low pressure is kinetically hindered, and isobaric experiments tend to yield the 9R structure upon temperature lowering.^61,62^ However, Ackland et al. showed, with careful calculations accounting for nuclear quantum effects, that fcc is indeed the true thermodynamic ground state, and they synthesized fcc Li at low pressure and temperature via a different pressure–temperature pathway (decompression).^56^ All together, the near energetic degeneracy of the close-packed polytypes and the unusual flatness of the associated energy landscape suggest the metal will be mechanically soft and prone to plastic deformation.^63^

Finally, the agreement between the OFDFT and KSDFT predictions for Li (Figure 2, top) is strong. We would not expect OFDFT to resolve perfectly the tiny energy differences between close-packed phases; even our KSDFT calculations likely have errors of some μeV/atom. However, OFDFT is clearly able to resolve energy differences nearer 1 meV/atom, evidenced by its correct prediction for bcc. To quantify how well OFDFT orders the structures by energy, we compute Spearman rank-order correlations between the OFDFT energies and the two varieties of KSDFT energies. A Spearman correlation of one indicates perfect agreement in the ranking of structures, while a value of zero indicates no association. The results are 0.82 and 0.79 when OFDFT is compared with KSDFT with local and nonlocal pseudopotentials, respectively. If all but one of the close-packed variations are omitted, acknowledging the near degeneracies and the limitations of our convergence criteria, the rank-order correlations improve to 1.00 and 0.98.

### Sodium

The results for Na (Figure 3) are somewhat similar to those for Li. The search yielded several close-packed polytypes differing by less than 1 meV/atom in energy, and the bcc structure, which is observed at room temperature, was found most frequently. However, the results also suggest that Na is more prone to small distortions of the close-packed motif. For example, the hcp structure and the two-atom *Cmcm* structure adjacent to it on the SHEAP map were located equally often, and the latter is a slight orthorhombic distortion of the former. Such structures have been hypothesized to explain the diffraction pattern observed when Na is cooled from room temperature.^64^ The fact that the *Cmcm* structure lies between bcc and hcp on the SHEAP map is consistent with this interpretation.

To the best of our knowledge, the true thermodynamic ground state of Na has not been determined unequivocally,^64−71^ and our calculations do not resolve this issue. Nevertheless, its flat energy landscape, like that of Li, suggests that Na metal will be mechanically soft and will exhibit fluidlike deformation behavior.^63^

Finally, the OFDFT performance (Figure 3, top) was excellent for Na. The Spearman rank-order correlation between the OFDFT predictions and the KSDFT predictions (with both local and nonlocal pseudopotentials) is 0.99.

### Magnesium

The results for Mg (Figure 4) are more straightforward than those for Li and Na. The lowest-energy structure is clearly hcp, which is also the structure observed at room temperature. It was found more often than any other closed-packed structure in the search. Related structures like *hhhhc* are roughly 1 meV/atom higher in energy, intermediate close-packed structures like *hhcc* are a few meV/atom higher in energy, and the fcc structure is a full 10 meV/atom higher in energy than hcp. Notably, bcc is absent from the Mg SHEAP map, consistent with prior observations that bcc Mg is unstable.^63,72^ However, the *I*4/*mmm* structure that was found a large number of times has body-centered tetragonal geometry with c/a ratio 1.34, between that of bcc (1.00) and fcc (1.41).

The OFDFT performance (Figure 4, top) was very good for Mg. The Spearman rank-order correlations between the OFDFT energies and those computed with KSDFT with local and nonlocal pseudopotentials are 0.99 and 0.98, respectively.

### Aluminum

The results for Al (Figure 5) are also fairly straightforward. The room-temperature fcc structure is the unambiguous ground state with a very high frequency of occurrence. Numerous close-packed polytypes appear, with the *hcc* (triple hcp) and *hchcc* structures slightly higher in energy than fcc, and others higher still. One interesting feature is the 11-atom P1̅ structure lying near fcc in the SHEAP map; it relaxed into fcc during the secondary optimization using KSDFT with the nonlocal pseudopotential.

The spread between the OFDFT and the KSDFT energies (Figure 5, top) is greater for Al than for the other elements (although, on a per electron basis, the Al results resemble the others). Nevertheless, the OFDFT energy ordering remains mostly correct, and the Spearman rank-order correlations are 0.95 and 0.81, respectively, between the OFDFT predictions and the KSDFT energies with local and nonlocal pseudopotentials. If the unstable P1̅ structure is omitted, both correlations improve to 0.97.

## Conclusions

The random structure searching approach to structure prediction is simple and general. To succeed, it requires little more than a robust, computationally efficient method for determining the enthalpy of a candidate structure. In this work, we established that OFDFT can serve this purpose, paving the way for future searches that are larger and more wide-ranging than feasible with conventional KSDFT. Importantly, while OFDFT proved accurate for the free-electron-like metals we considered—and less expensive than KSDFT, which would have required dense Brillouin zone sampling—it need not yield perfect predictions to excel as an engine for random structure searching. The key attribute is broad-based reliability: if OFDFT correctly identifies the salient basins in an energy landscape, it succeeds. Refinement of results with KSDFT adds little extra cost.

Our searching explored the landscapes of Li, Na, Mg, and Al at zero pressure. For Li and Na, the striking results were the many close-packed structures of nearly identical energy. These findings—bolstered by the fact that their room-temperature structure, bcc, is higher in energy by only one or two meV/atom—suggest mechanical softness and ease of plastic deformation. By contrast, the searching for Mg and Al revealed more definitive low-temperature ground state structures, identical with those observed at room temperature. Importantly, the bcc structure was *not* found for Mg or Al, consistent with calculations suggesting it would be unstable.

Looking ahead, we anticipate a mutually beneficial relationship between OFDFT and random structure searching, just as the latter has strengthened machine-learned interatomic potentials.^73,74^ Where OFDFT is presently accurate, its low cost will enable structure searching over wide ranges of compositions and pressures. At the same time, the demands of random search will spur new developments in OFDFT. During kinetic energy functional or local pseudopotential construction, a common workflow involves preselecting a modest set of structures for benchmarking, exactly as we did in the Methods. However, this philosophy is imperfect, or at least incomplete—what if the chosen structures are not the most relevant ones? The stochastic nature, exploratory qualities, and comprehensiveness of random structure searching make it a harsh but fruitful test.

## Supporting Information Available

Equilibrium volumes and bulk moduli for OFDFT benchmarking, additional information on the SHEAP maps, and assessment of the exchange-correlation functional (PDF)
