# Supplementary material for: Random Structure Searching with Orbital-Free Density Functional Theory
Source: J Phys Chem A. 2021 Feb 15;125(7):1650–60. doi: 10.1021/acs.jpca.0c11030 (PMC8023714; doi:10.1021/acs.jpca.0c11030)
Supplement: Supplementary file 1 — jp0c11030_si_001.pdf [file jp0c11030_si_001.pdf]

Supplemental Information: Random Structure Searching with Orbital-free Density Functional Theory

William C. Witt,[*,†] Benjamin W. B. Shires,[†] Chuin Wei Tan,[‡] Wojciech J. Jankowski,[¶] and Chris J. Pickard[*,†,§]

†*Department of Materials Science and Metallurgy, University of Cambridge, Cambridge, United Kingdom*

‡*Downing College, University of Cambridge, Cambridge, United Kingdom*

¶*Trinity College, University of Cambridge, Cambridge, United Kingdom*

§*Advanced Institute for Materials Research, Tohoku University, Sendai, Japan*

E-mail: wcw28@cam.ac.uk; cjp20@cam.ac.uk

Preliminary Benchmarking: Equilibrium Volumes and Bulk Moduli

Tables S1 and S2 show the equilibrium volumes and bulk moduli corresponding to the relative energies in Table 1 of the main paper.

Table S1: Equilibrium volumes (Å^3/atom) for several elements and crystal structures as predicted by OFDFT using LPPs and eight variations of the same KE functional (Wang-Teter, Perrot, Smargiassi-Madden, and Wang-Govind-Carter, along with their exponential-stabilized forms) and as predicted by KSDFT using both LPPs (KS-L) and NLPPs (KS-NL). The italicized rows hold the optimized c/a ratios corresponding to the hcp structures.

	WT	WT-e	P	P-e	SM	SM-e	WGC	WGC-e	KS-L	KS-NL
**Li**										
fcc	20.2	20.3	20.3	20.4	19.9	20.0	20.2	20.3	20.2	20.2
hcp	20.3	20.3	20.4	20.4	19.9	20.0	20.2	20.3	20.2	20.3
c/a	*1.63*	*1.63*	*1.63*	*1.63*	*1.63*	*1.63*	*1.63*	*1.63*	*1.63*	*1.63*
bcc	20.2	20.2	20.3	20.3	19.9	19.9	20.2	20.2	20.2	20.3
sc	21.9	22.0	22.0	22.1	21.6	21.6	21.9	21.9	21.9	20.4
cd	30.3	29.8	30.2	29.8	30.8	30.1	30.4	29.8	30.5	25.6
**Na**										
	WT	WT-e	P	P-e	SM	SM-e	WGC	WGC-e	KS-L	KS-NL
fcc	37.1	37.1	37.2	37.3	36.7	36.8	37.1	37.1	37.1	37.1
hcp	37.1	37.2	37.3	37.3	36.8	36.8	37.1	37.1	37.1	37.2
c/a	*1.63*	*1.63*	*1.63*	*1.63*	*1.63*	*1.63*	*1.63*	*1.63*	*1.63*	*1.63*
bcc	37.0	37.1	37.2	37.2	36.7	36.7	37.0	37.1	37.0	37.1
sc	39.7	39.7	39.8	39.8	39.4	39.4	39.7	39.7	39.6	39.8
cd	53.0	51.7	52.8	51.7	54.4	52.3	53.2	51.9	53.4	54.8
**Mg**										
	WT	WT-e	P	P-e	SM	SM-e	WGC	WGC-e	KS-L	KS-NL
hcp	23.1	23.2	23.4	23.5	21.5	21.9	23.0	23.1	22.9	22.9
c/a	*1.63*	*1.63*	*1.63*	*1.63*	*1.63*	*1.63*	*1.63*	*1.63*	*1.63*	*1.62*
fcc	23.2	23.3	23.6	23.6	21.8	22.1	23.2	23.3	23.1	23.1
bcc	23.0	23.1	23.4	23.4	21.4	21.8	22.9	23.0	22.8	22.9
sc	27.2	27.1	27.4	27.3	26.5	26.5	27.2	27.1	27.1	27.5
cd	39.5	37.8	40.1	38.0	41.8	38.2	40.0	38.0	39.9	40.3
**Al**										
	WT	WT-e	P	P-e	SM	SM-e	WGC	WGC-e	KS-L	KS-NL
fcc	16.8	16.8	17.0	17.0	15.7	15.8	16.7	16.8	16.6	16.5
hcp	16.9	16.9	17.1	17.1	15.8	15.9	16.8	16.9	16.7	16.6
c/a	*1.66*	*1.66*	*1.66*	*1.66*	*1.66*	*1.66*	*1.66*	*1.67*	*1.64*	*1.66*
bcc	17.2	17.2	17.4	17.4	16.0	16.2	17.1	17.1	17.0	16.9
sc	19.9	19.7	20.1	19.9	19.6	19.3	19.9	19.7	19.9	20.1
cd	28.8	27.2	-	27.8	28.8	26.9	29.3	27.4	27.3	27.6

Table S2: Bulk moduli (GPa) for several elements and crystal structures as predicted by OFDFT using LPPs and eight variations of the same KE functional (Wang-Teter, Perrot, Smargiassi-Madden, and Wang-Govind-Carter, along with their exponential-stabilized forms) and as predicted by KSDFT using both LPPs (KS-L) and NLPPs (KS-NL)

	WT	WT-e	P	P-e	SM	SM-e	WGC	WGC-e	KS-L	KS-NL
**Li**										
fcc	16	16	16	16	16	16	16	16	16	14
hcp	16	16	16	16	16	16	16	16	16	17
bcc	16	16	16	16	16	16	16	16	16	14
sc	12	12	12	12	13	13	12	12	12	12
cd	6	7	6	7	5	6	6	7	6	5
**Na**										
fcc	7.2	7.2	7.2	7.2	7.2	7.3	7.2	7.2	7.2	7.7
hcp	7.2	7.2	7.2	7.2	7.3	7.3	7.2	7.2	7.2	7.7
bcc	7.2	7.2	7.2	7.2	7.3	7.3	7.2	7.2	7.3	7.8
sc	5.9	5.9	5.9	5.9	5.8	5.8	5.8	5.9	5.8	6.2
cd	2.9	3.3	2.9	3.3	2.5	3.1	2.8	3.2	2.9	3.0
**Mg**										
hcp	37	37	37	37	39	40	37	38	39	36
fcc	37	37	36	37	38	39	37	37	38	35
bcc	37	37	36	36	39	40	37	37	38	35
sc	24	24	24	25	22	23	24	24	24	23
cd	11	14	11	14	9	13	11	14	10	11
**Al**										
fcc	79	80	77	78	82	85	79	80	77	79
hcp	77	78	75	76	80	83	77	78	75	75
bcc	71	73	69	71	75	77	71	73	71	70
sc	57	62	56	61	53	61	57	61	58	56
cd	23	35	-	30	28	38	20	34	39	38

The SHEAP Maps: Additional Information

As described in the main paper, the SHEAP maps are based on SOAP descriptors for the structures. We employed SOAP parameters of $r_{\mathrm{cut}} = 5$, $n_{\mathrm{max}} = 15$, $l_{\mathrm{max}} = 9$, and $\sigma = 0.5$. We classified two structures as identical if the norm of their SOAP difference vector fell below a threshold of 0.7/0.25/0.2/0.07 for Li/Na/Mg/Al, respectively. For the SHEAP algorithm itself, we used a perplexity of 30 and a minimum hard sphere radius of 0.01 when creating the images. (For the three-dimensional SHEAP visualizations discussed next, we used the same parameters aside from a minimum hard sphere radius of 0.015.)

Dimensionality reduction for data visualization is not restricted to two map dimensions. To complement the two-dimensional SHEAP maps (Figs. 2-5 in the main paper), we generated corresponding three-dimensional SHEAP maps, which are summarized in Fig. S1. These results show that two map dimensions are sufficient for capturing the salient features in the data. In fact, the three-dimensional SHEAP maps are nearly flat, with little along the third dimension aside from the depth of the spheres. Our general experience with SHEAP suggests that, often, a third dimension becomes beneficial only with larger data sets.

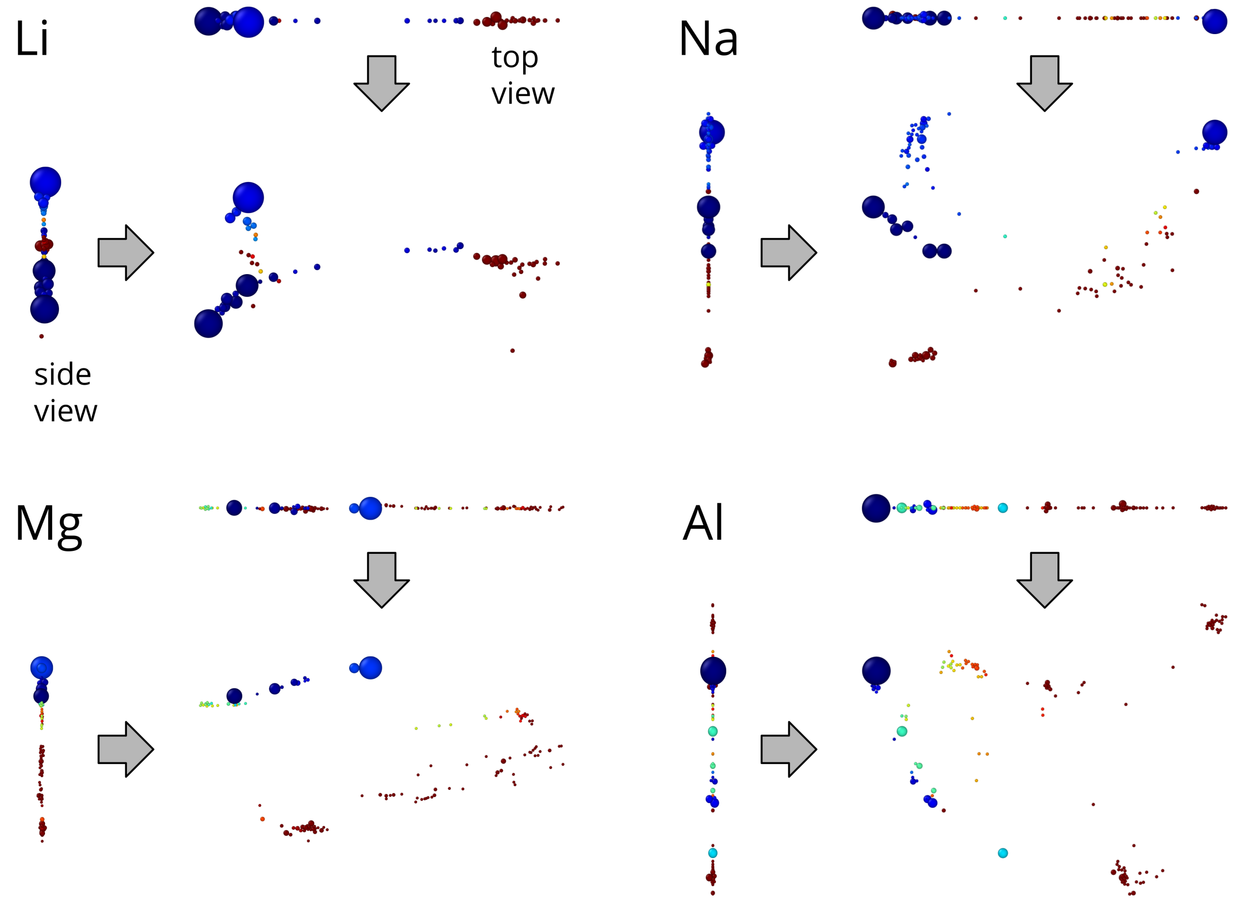

Figure S1: Three-dimensional SHEAP maps corresponding to the two-dimensional SHEAP maps found in Figs. 2-5 of the main paper. The maps are mostly flat, as seen from the side and top views, indicating that two dimensions are sufficient for representing the main features.

Assessing the Exchange-Correlation Functional

To build confidence that the PBE exchange-correlation functional is suitable for the nearly-free-electron metals considered, we repeated some geometry optimizations with the LDA and PBEsol functionals. Beginning with the OFDFT-derived structures from the top portions of Figs. 2-5 in the main paper, we re-relaxed them using KSDFT and both LDA and PBEsol. Exactly as with PBE, these relaxations were performed with CASTEP using C19 nonlocal pseudopotentials generated on-the-fly for the particular exchange-correlation functional.

The results are summarized in Figs. S2-S5. They show that the relative energies, even after geometry optimization, are insensitive to the choice of exchange-correlation functional. Frequently, the three lines representing the nonlocal pseudopotential calculations (for LDA, PBE, and PBEsol) are indistinguishable on the scale of the plots.

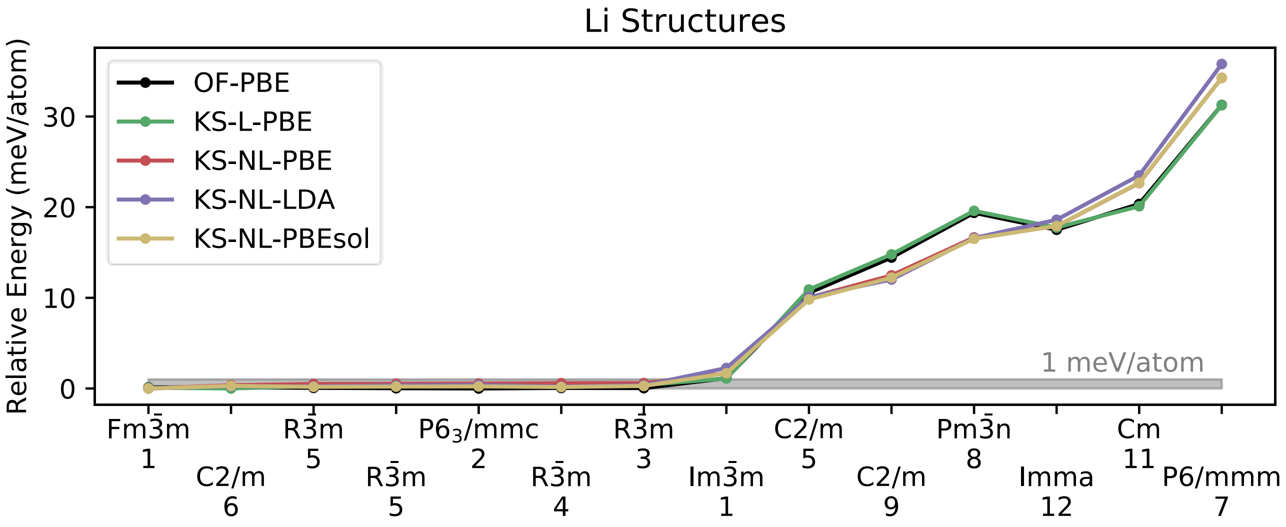

Figure S2: Relative energies of a subset of Li structures computed with OFDFT (OF) and KSDFT with local or nonlocal pseudopotentials (KS-L and KS-NL, respectively). The labels also include the exchange-correlation functional used for the final geometry optimization (LDA, PBE, or PBEsol). Compare with Fig. 2 (*top*) in the main paper.

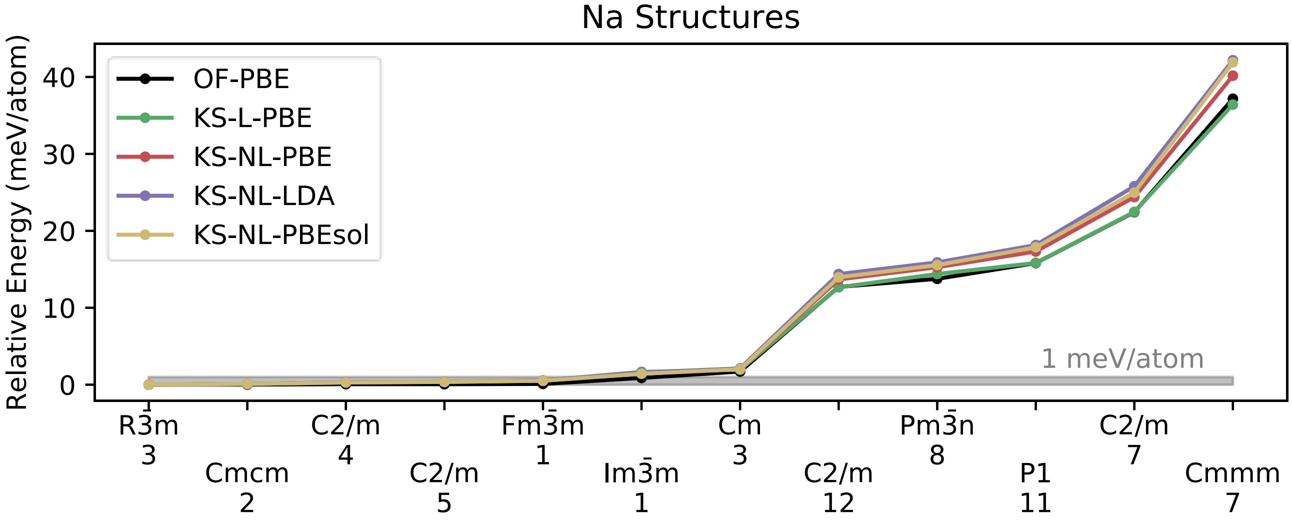

Figure S3: Relative energies of a subset of Na structures computed with OFDFT (OF) and KSDFT with local or nonlocal pseudopotentials (KS-L and KS-NL, respectively). The labels also include the exchange-correlation functional used for the final geometry optimization (LDA, PBE, or PBEsol). Compare with Fig. 3 (*top*) in the main paper.

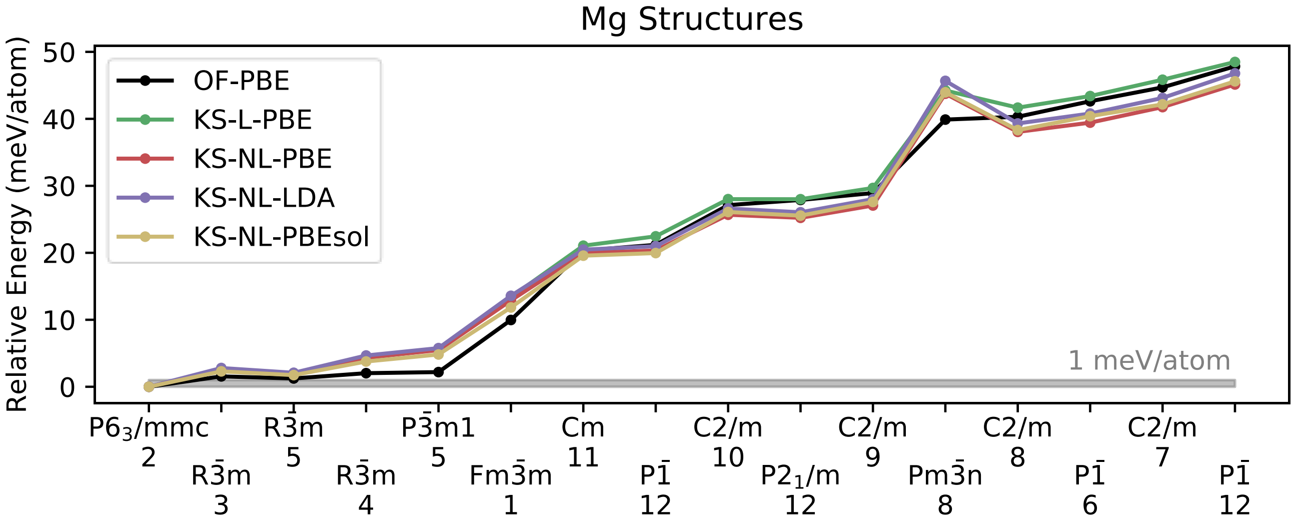

Figure S4: Relative energies of a subset of Mg structures computed with OFDFT (OF) and KSDFT with local or nonlocal pseudopotentials (KS-L and KS-NL, respectively). The labels also include the exchange-correlation functional used for the final geometry optimization (LDA, PBE, or PBEsol). Compare with Fig. 4 (*top*) in the main paper.

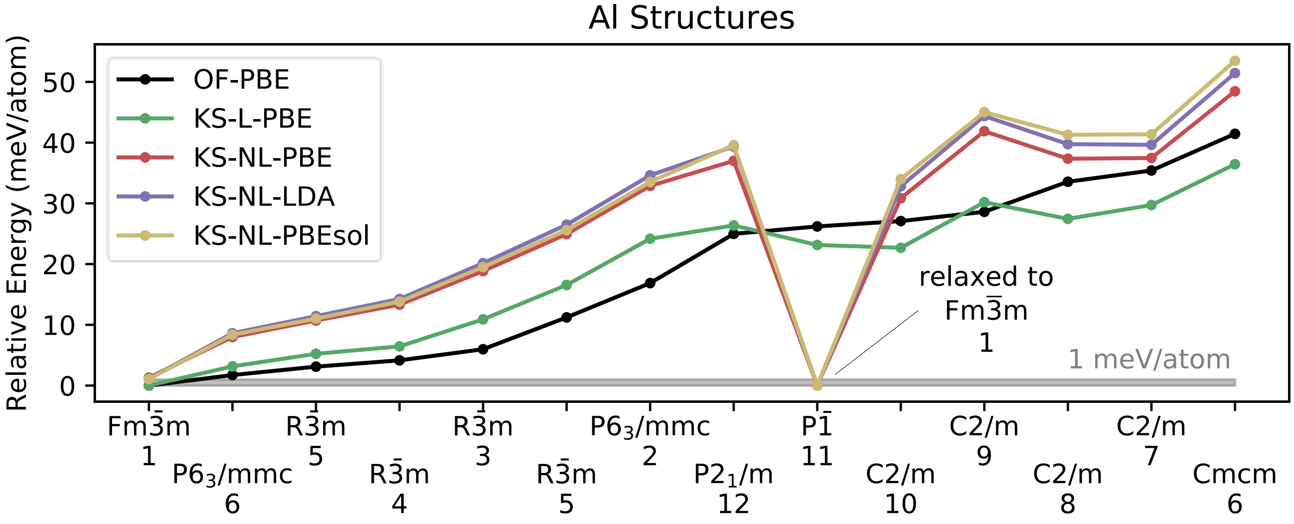

Figure S5: Relative energies of a subset of Al structures computed with OFDFT (OF) and KSDFT with local or nonlocal pseudopotentials (KS-L and KS-NL, respectively). The labels also include the exchange-correlation functional used for the final geometry optimization (LDA, PBE, or PBEsol). Compare with Fig. 5 (*top*) in the main paper.
